# Supplementary material for: The Construction and Exploration of a Comprehensive MicroRNA Centered Regulatory Network in Foxtail Millet (Setaria italica L.)
Source: Front Plant Sci. 2022 May 6;13:848474. doi: 10.3389/fpls.2022.848474 (PMC9121102; doi:10.3389/fpls.2022.848474)
Supplement: Supplementary file 3 [file Data_Sheet_3.zip › Sit-miRN3000_Seita.7G057600.1_1210_TPlot.pdf]

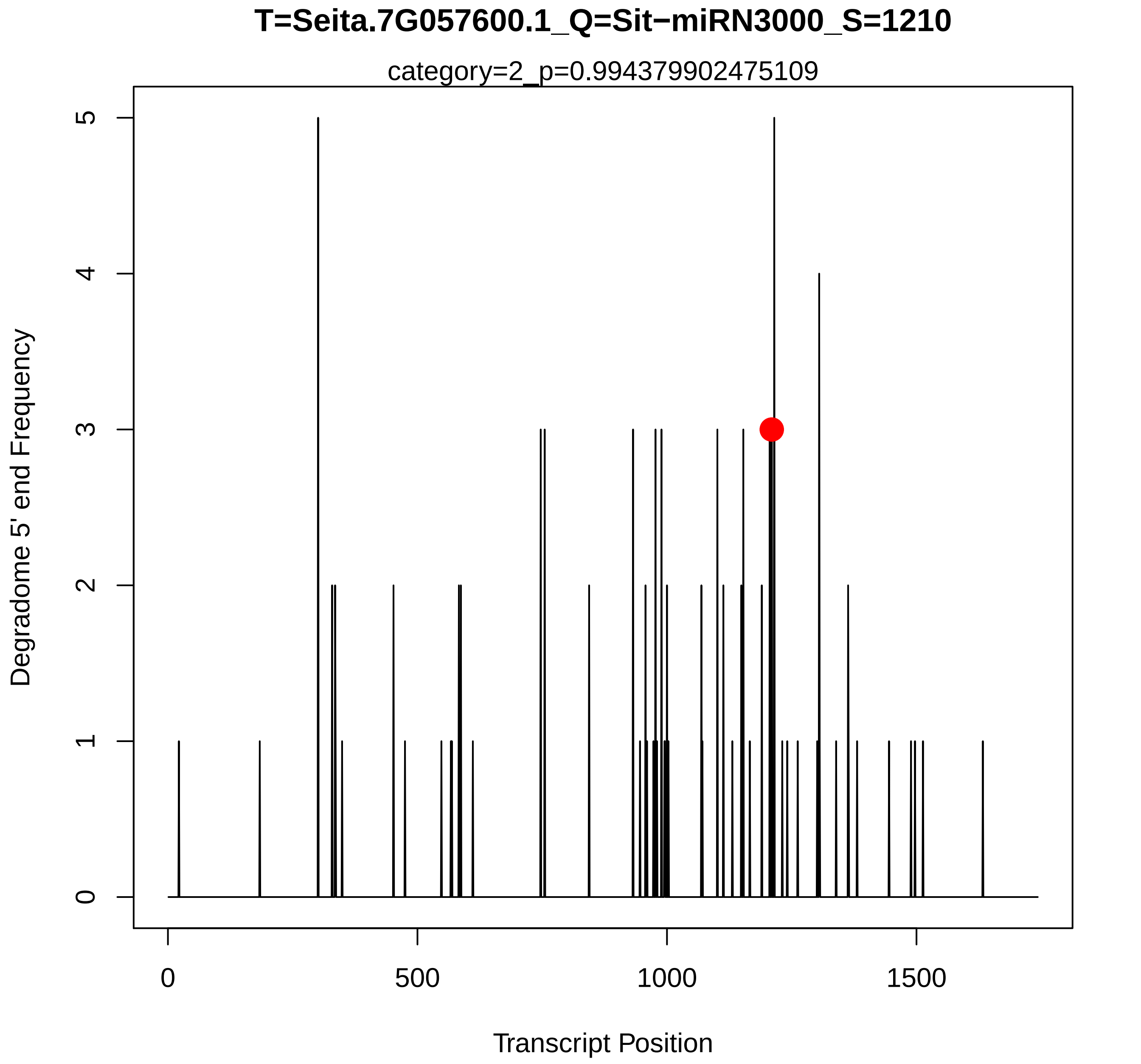

T=Seita.7G057600.1_Q=Sit−miRN3000_S=1210
category=2_p=0.994379902475109
Degradome 5' end Frequency
0
1
2
3
4
5
0
500
1000
1500
Transcript Position
